# White Matter Abnormalities in Patients With Typhoon-Related Posttraumatic Stress Disorder

**DOI:** 10.3389/fnhum.2021.665070

**Published:** 2021-09-29

**Authors:** Hui Juan Chen, Rongfeng Qi, Jun Ke, Jie Qiu, Qiang Xu, Yuan Zhong, Guang Ming Lu, Feng Chen

**Affiliations:** ^1^Department of Radiology, Hainan General Hospital (Hainan Affiliated Hospital of Hainan Medical University), Haikou, China; ^2^Department of Medical Imaging, Jinling Hospital, Medical School of Nanjing University, Nanjing, China; ^3^Department of Radiology, The First Affiliated Hospital of Soochow University, Suzhou, China; ^4^Department of Ultrasound, Hainan General Hospital (Hainan Affiliated Hospital of Hainan Medical University), Haikou, China

**Keywords:** posttraumatic stress disorder, diffusion tensor imaging, fractional anisotropy, mean diffusivity, axial diffusivity, radial diffusivity

## Abstract

Patients with posttraumatic stress disorder (PTSD) might have white matter abnormalities. However, less is known about white matter changes after exposing a specific traumatic event. The purpose of this study was to explore the abnormalities of diffusion in cerebral white matter and its relationship with the clinical symptoms in patients with PTSD by using diffusion tensor imaging (DTI). Diffusion-weighted imaging of the cerebrum was performed in typhoon survivors with (*n* = 27) and without PTSD (*n* = 33) and healthy controls (HCs) (*n* = 30). Differences in fractional anisotropy (FA), mean diffusivity (MD), axial diffusivity (AD), and radial diffusivity (RD) were calculated among groups using voxel-based analysis of the DTI data. Correlations between diffusion indices and clinical symptoms in patients with PTSD were also assessed. Both patients with PTSD and trauma-exposed control (TEC) group showed increased FA in the anterior limb of the internal capsule, forceps of the corpus callosum, and corona radiata relative to the HC group. Additionally, there was a negative correlation between FA values in the white matter and the clinical symptoms. Trauma exposure may result in disruption of cerebral white matter in individuals with or without PTSD, particularly in the frontal fibers. Aberrant white matter alterations may be associated with the severity of PTSD symptoms.

## Introduction

Posttraumatic stress disorder (PTSD) is a severe anxiety disorder that develops following a traumatic event that involves the threat of death or serious injury to oneself or others. It mainly includes four symptom clusters, namely, reexperience, avoidance, negative cognitions and mood, and arousal. The lifetime prevalence of PTSD in the American general population is high, with an estimated rate of 6.8% (Kessler et al., 2005). Importantly, PTSD is usually comorbid with additional debilitating symptoms, such as depression and substance abuse. Therefore, a better understanding of the neurobiological dysfunctions that underline PTSD might be helpful in the development of improved diagnostic and therapeutic approaches.

It is reported that PTSD is associated with white matter abnormalities (Siehl et al., 2018). However, these results have been inconsistent. For example, some studies found decreased fractional anisotropy (FA) in the corpus callosum (Lei et al., 2015; Rinne-Albers et al., 2016; O'Doherty et al., 2018), prefrontal cortex (PFC) (Schuff et al., 2011), anterior cingulum (Zhang et al., 2011; Ju et al., 2020), and posterior cingulum (Fani et al., 2012), while others found increased FA in the posterior cingulum (Zhang et al., 2012; Weis et al., 2018) and superior frontal gyrus (Zhang et al., 2011).

The following reasons may account for the inconsistency of the results. First, the sample size was relatively small in earlier studies, which limit the persuasion of the results (Zhang et al., 2011, 2012; Fani et al., 2012). Second, manual tracing analysis in predefined regions of interest (ROIs) which largely depend on specific prior hypotheses was applied in most early white matter studies (Daniels et al., 2013). Whole-brain voxel-based analysis may overcome this disadvantage. Third, most previous studies only compared patients with PTSD with nontraumatized healthy controls (HCs) or only trauma-exposed controls (TECs). This made it difficult to differentiate whether alterations were associated with PTSD *per se* or simply with traumatic stress exposure (Li et al., 2014). Only a few studies have done the comparison of patients with PTSD with both HCs and TECs who experienced the same trauma but did not develop PTSD (O'Doherty et al., 2018; Siehl et al., 2020). Finally, the trauma type and intensity may significantly differ among individuals. Studies including individuals with/without PTSD who were exposed to the same traumatic event and HCs and using voxel-based approaches may provide more information regarding the mechanism of PTSD.

Diffusion-weighted imaging (DWI) is a noninvasive tool for estimating white matter integrity (Hess and Mukherjee, 2007). By measuring the properties such as FA, it could discover early neuropathological alterations. Decreased FA might be a reflection of damage or degeneration in white matter. Additionally, mean diffusivity (MD) that assesses general diffusion of water molecules has been accepted as a property of microstructural architecture integrity. Unlike the ROI method, the voxel-based diffusion tensor analysis (VBA) could provide more objective and reliable results without prior knowledge (Abe et al., 2006).

This study used DWI to explore whole-brain microstructural alterations of white matter in patients with PTSD in comparison to both trauma-naive and trauma-exposed individuals without PTSD. Our previous functional magnetic resonance imaging (fMRI) studies found altered function in the prefrontal-limbic system (Chen et al., 2019, 2021). However, structural alterations underlying the white matter alterations had not been investigated. Based on the previous studies, we hypothesized that there are white matter abnormalities in patients with PTSD such as cingulum and corpus callosum and that they are associated with PTSD symptomatology.

## Materials and Methods

### Participants and Clinical Assessment

A category of five super typhoon named Rammasun stuck in Wenchang City in Hainan Province in July 2014. The residents here were greatly influenced by this typhoon. A minimum of 14 people lost their lives due to this typhoon. Particularly, Loudoun Farm of Wenchang City was most affected by this damaging typhoon. Seventy typhoon-exposed subjects were recruited from this area including 36 with PTSD (i.e., 9 males and 27 females) and 34 without PTSD (i.e., TEC, 7 males, and 27 females), who were all screened with the PTSD Checklist-Civilian Version (PCL). This study was in line with the declaration of Helsinki and was approved by the Ethics Committee of Hainan General Hospital and the Second Xiangya Hospital of Central South University. All participants provided written informed consent after a detailed description of this study. The PCL is a 17-item self-report instrument that could evaluate the severity of DSM-IV-defined PTSD symptoms. The diagnosis of PTSD was made according to DSM-IV diagnostic criteria for current PTSD, and symptoms were further evaluated by the Clinician-Administered PTSD Scale (CAPS) (Weathers et al., 2001). The CAPS assesses 17 core PTSD symptoms listed in the DSM-IV and obtains information regarding symptom onset, duration, and functional impact. The Structured Clinical Interview for DSM-IV was used for the determination regarding the absence or presence of other psychiatric disorders. Additionally, 32 HCs (i.e., 9 males and 23 females) who did not meet DSM-IV Criterion A1 for PTSD were recruited *via* advertisement from Haikou. The Self-Rating Anxiety Scale (SAS) (Zung, 1971) and Self-Rating Depression Scale (SDS) (Zung, 1965) were used to evaluate anxiety and depression status, respectively.

The exclusion criteria were as follows: age <18 years or >65 years, left handedness, previous history of head injury or loss of consciousness, significant medical and neurological conditions, comorbid lifetime or current psychiatric disorders other than depression and anxiety, alcohol or drug abuse/dependence, use of psychiatric medication, and contraindications to MRI such as claustrophobia, pregnancy, and ferromagnetic implants. In the PTSD group, complete imaging data were not available for three female subjects, and six were removed for the following reasons: denture-related artifacts (one female and one male), brain infarction revealed by conventional MRI (one female), pregnancy (one female), and excessive movement (translation >1.5 mm or rotation >1.5° at any direction) during MRI scanning (one male and one female). Additionally, we excluded one female TEC for excessive movement and two male HCs for brain infarction. Thus, 27 patients with PTSD, 33 TECs, and 30 HCs were ultimately included in the statistical analysis.

### MRI Data Acquisition

The structural MRI was acquired at Hainan General Hospital using a 3 Tesla MR scanner (Skyra, Siemens Medical Solutions, Erlangen, Germany) equipped with a 32-channel standard head coil. The heads of subjects were immobilized using a foam pad and a Plexiglas head cradle. DWI data, with 30 noncollinear directions (*b* = 1,000 s/mm^2^), as well as a reference image without diffusion weighting gradient (*b* = 0), were acquired using a single-shot spin-echo echo-planar image (SE-EPI) sequence. Array spatial sensitivity encoding was used to reduce susceptibility and eddy-current artifacts. The DWI data were obtained using the following parameters: TR/TE = 9,000/90 ms, matrix = 128 × 128 mm^2^, FOV = 256 × 256 mm^2^, and slice thickness = 2 mm. The sections were placed approximately parallel to the anterior commissure-posterior commissure line.

### Image analysis

Diffusion tensor imaging (DTI) data were performed using the standard procedure using PANDA software version 1.3.0 developed by Cui et al. (2013). The processing procedures include skull stripping, correcting the eddy current distortions by registering the DW images to a b0 image with an affine transformation. Then, voxel-wise maps of FA, MD, axial diffusivity (AD), and radial diffusivity (RD) were calculated using the DTIFIT program. For each subject, individual FA images of native space were first registered to the FA template (FMRIB58_FA template) in the Montreal Neurological Institute (MNI) space, and then the resultant warping transformations were applied to write the images of the diffusion metrics (i.e., FA, MD, AD/L1, and RD/L23m) into the MNI space, with each voxel being 2 × 2 × 2 mm^3^. The normalized images were smoothed using an isotropic Gaussian kernel (6-mm full width at half maximum) for statistical analysis.

Then, we conducted the white matter voxel-wise analysis using SPM8 software (http://www.fil.ion.ucl.ac.uk/spm/) running in MATLAB 2012a (MathWorks, Natick, Mass, USA).

### Statistical Analysis

The chi-squared test was used to analyze gender distribution, and one-way ANOVA was performed for all continuous variables except for PCL scores, for which an independent *t*-test was used to examine differences between the PTSD group and the TEC group. The above analyses were conducted using SPSS version 21.0 (SPSS Inc., Chicago, IL, USA), with the significance threshold set at *P* < 0.05. SPM8 was used to analyze the FA, MD, AD, and RD differences among the three groups by using ANCOVA with age, sex, and education level as covariates, followed by *post-hoc t*-tests to examine the between-group differences. We evaluated brain white matter by using a thresholded mean FA image (FA ≥ 0.2) for masking white matter. This generated mask was also used for the MD, RD, and AD analyses. For the FA and MD analyses, *post-hoc t*-tests were corrected (*P* < 0.05) for multiple comparisons using the Gaussian random field theory (Worsley et al., 2004) with the voxel level set at *P* < 0.01 and the cluster level set at *P* < 0.05 (cluster size > 20 voxels). The JHU-White-Matter-labels atlas was used to label each cluster. To investigate the association between the severity of PTSD symptoms and brain measures, FA, MD, AD, and RD values with significant group differences were extracted and then correlated against the CAPS total scores, PCL scores, and SAS and SDS by using Pearson correlation analysis in the PTSD group. The correlation analysis was accomplished using SPSS, with a significant threshold of *P* < 0.05 (not corrected).

## Results

### Demographic and Clinical Variables

The demographic and clinical characteristics are summarized in Table 1. There was no significant difference in age (*F* = 0.317, *P* = 0.729) and gender distribution (*P* = 0.912) among the PTSD, TEC, and HC groups. There was a significant difference in the education level among the three groups (*F* = 8.396, *P* < 0.001). *Post-hoc* analyses demonstrated that the education level of the HC group was higher than that in the PTSD group (*P* < 0.001) and the TEC group (*P* = 0.001). No significant difference was found between the PTSD and TEC groups (*P* = 0.518) for the education level. The PCL scores were statistically higher in the PTSD group relative to the TEC group (*P* < 0.001). Ten patients with PTSD had current psychiatric comorbidity: nine with depression (two males and seven females) and one with anxiety disorder (1 female). Significant differences were also found among the three groups in the SAS (*F* = 81.864, *P* < 0.001) and SDS scores (*F* = 101.915, *P* < 0.001). *Post-hoc* analyses showed that the TEC group had significantly higher scores for the SAS (*P* = 0.025) and SDS (*P* = 0.003) groups than those in the HC group but was significantly lower when comparing with the PTSD group (all *P* < 0.001).

**Table 1:** Demographic and clinical data of traumatized individuals and healthy controls.

	**PTSD (*****n*** **= 27)**	**TEC (*****n*** **= 33)**	**HC (*****n*** **= 30)**	***P*** **-value**
Gender (males/females)	7/20	7/26	7/23	0.912^a^
Age (year)	48.4 ± 10.3	48.5 ± 7.5	49.9 ± 6.1	0.729^b^
Education (year)	6.4 ± 3.4	7.0 ± 3.4	9.7 ± 3.3	<0.001^b^
SAS score	65.8 ± 13.3	41.3 ± 8.1	36.0 ± 5.5	<0.001^b^
SDS score	69.6 ± 13.2	41.3 ± 9.1	33.5 ± 7.2	<0.001^b^
PCL score	53.7 ± 8.5	28.9 ± 5.4		<0.001^c^
CAPS total score	78.2 ± 19.3			

a *P-value obtained using the chi-square test*;

b *P-value obtained with one-way ANOVA; and*

c *P-value obtained using the independent t-test for continuous variables. Values are given as mean ± SD except for gender, which is presented as a number. PTSD, posttraumatic stress disorder; TEC, trauma-exposed control; HC, healthy control; SAS, Self-Rating Anxiety Scale; SDS, Self-Rating Depression Scale; PCL, PTSD Checklist; CAPS, Clinician-Administered PTSD Scale*.

### Voxel-Based Analysis of Diffusion Properties

**Figure 1:**
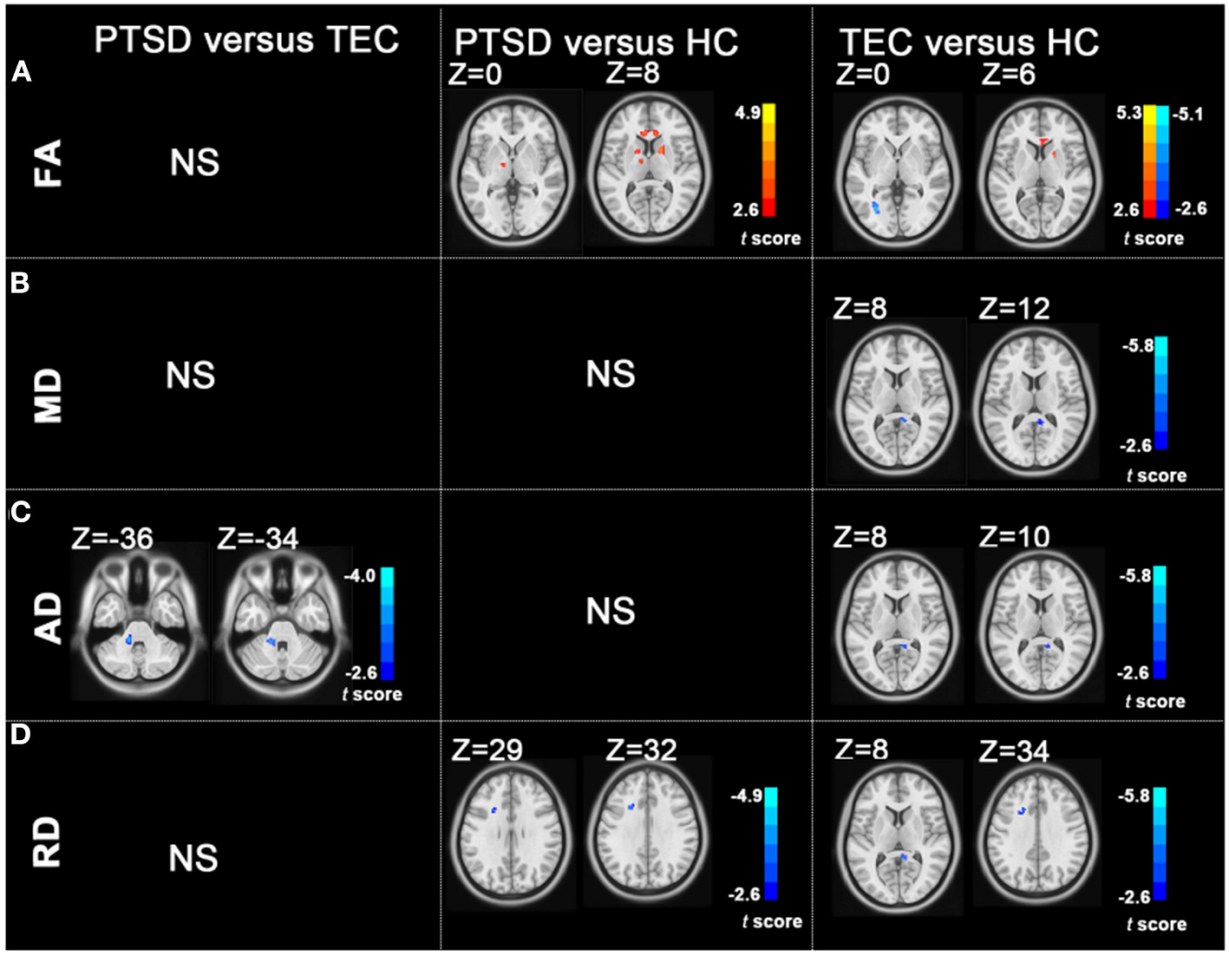
Group differences in FA, MD, AD, and RD revealed by using *post-hoc* tests **(A–D)**. **(A)** The PTSD group showed increased FA values in the left posterior limb of the internal capsule, bilateral anterior limb of the internal capsule, right superior longitudinal fasciculus, bilateral anterior corona radiata, and right posterior corona radiata relative to the HC group; the TEC group showed decreased FA values in the left posterior corona radiata and increased FA values in the right anterior limb of the internal capsule, right posterior corona radiata, and bilateral anterior corona radiata relative to the HC group. **(B)** The TEC group had decreased MD values in the right cingulum relative to the HC group. **(C)** The PTSD group had decreased AD in the left middle cerebellar peduncle compared with the TEC group. The TEC group had decreased AD in the right cingulum relative to the HC group. **(D)** Patients with PTSD had decreased RD in the left anterior corona radiata compared with the HC group. The TEC group had decreased RD in the right cingulum, right inferior fronto-occipital fasciculus, right inferior longitudinal fasciculus, and left anterior corona radiata relative to the HC group (*P* < 0.05, GRF corrected). PTSD, posttraumatic stress disorder; TEC, trauma-exposed controls; HC, healthy controls; FA, fractional anisotropy; MD, mean diffusivity; AD, axial diffusivity; RD, radial diffusivity; GRF, Gaussian Random-Field.

The *t*-maps of the significantly altered FA, MD, AD, and RD were demonstrated in the coordinates of MNI space. *Post-hoc* analysis revealed that no statistical FA difference was found between the PTSD and the TEC groups. Patients with PTSD had increased FA values in the left posterior limb of the internal capsule, bilateral anterior limb of the internal capsule, right superior longitudinal fasciculus, bilateral anterior corona radiata, bilateral forceps of the corpus callosum, and right posterior corona radiata relative to the TEC group. In comparison with the HC group, TEC patients showed decreased FA values in the left posterior corona radiata and increased FA values in the right anterior limb of the internal capsule, right forceps of the corpus callosum, right posterior corona radiata, and bilateral anterior corona radiata (Table 2 and Figure 1).

**Table 2:** Brain regions showing white matter differences among the PTSD group, the TEC group, and the HC group.

	**Brain region**	**Voxel**	**MNI coordinates (*x*, *y*, *z*)**	**Peak *t*-score**
**FA**				
*PTSD vs. TEC*	None			
*PTSD vs. HC*				
	Posterior_limb_of_internal_capsule_L	81	−14, −6, −4	3.7
	Anterior_limb_of_internal_capsule_R	77	16, 8, 8	3.9
	Anterior_limb_of_internal_capsule_L	62	−18, 10, 2	4.1
	Superior longitudinal fasciculus R	88	40, −26, 28	3.3
	Forceps of Corpus Callosum R	43	8, 32, 8	3.3
	Forceps of Corpus Callosum L	38	−6, 32, 8	3.5
	Posterior_corona_radiata_R	68	18, −52, 22	3.8
	Anterior_corona_radiata_L	59	−24, 16, 30	3.6
	Anterior_corona_radiata_R	49	26, 24, 24	3.3
*TEC vs. HC*				
	Posterior thalamic radiation (include optic radiation) L	57	−30, −66, 0	−4.7
	Forceps of Corpus Callosum R	38	2, 24, 6	3.0
	Anterior_limb_of_internal_capsule_R	30	20, 10, 6	3.1
	Posterior_corona_radiata_R	37	26, −48, 24	3.3
	Anterior_corona_radiata_R	21	26, 18, 30	3.4
	Anterior_corona_radiata_L	70	−22, 16, 32	3.7
**MD**				
*PTSD vs. TEC*				
	None			
PTSD vs. HC	None			
*TEC vs. HC*				
	Cingulum (cingulate gyrus) R	96	8, −42, 8	−3.9
**AD**				
*PTSD vs. TEC*				
	Middle cerebellar peduncle L	96	−14, −38, −36	−3.6
*PTSD vs. HC*				
	None			
*TEC vs. HC*				
	Cingulum(cingulate gyrus) R	25	10, −44, 8	−3.6
**RD**				
*PTSD vs. TEC*				
	None			
*PTSD vs. HC*				
	Frontal white matter (Anterior_corona_radiata_L)	39	−24, 16, 32	−3.6
*TEC vs. HC*				
	Cingulum(cingulate gyrus) R	94	6, −40, 8	−4.1
	Frontal white matter (Anterior_corona_radiata_L)	53	−22, 16, 34	−3.7

*PTSD, posttraumatic stress disorder; TEC, trauma-exposed controls; HC, healthy control; MNI, Montreal Neurological Institute; FA, fractional anisotropy; MD, mean diffusivity; AD, axial diffusivity; RD, radial diffusivity*.

The PTSD group had no statistical MD values compared with the TEC group. No statistical MD difference was found between the PTSD and the HC groups. The TEC group had decreased MD values in the right cingulum (Table 2 and Figure 1).

The PTSD group had decreased AD in the left middle cerebellar peduncle compared with the PTSD group. No statistical AD difference was found between the PTSD and the HC groups. The TEC group had decreased AD in the right cingulum relative to the HC group (Table 2 and Figure 1).

No statistical RD difference was found between the PTSD and the TEC groups. Patients with PTSD had decreased RD in the left anterior corona radiata compared with the HC group. The TEC group had decreased RD in the right cingulum and left anterior corona radiata (Table 2 and Figure 1).

### Correlation Results

**Figure 2:**
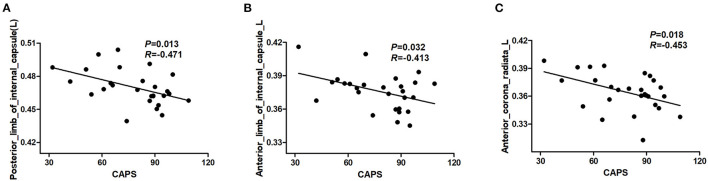
Results of correlation analyses between abnormal FA regions and severity of PTSD symptom in individuals with PTSD. The CAPS score was negatively correlated with the FA value of the left posterior limb of the internal capsule **(A)**. The CAPS score was negatively correlated with the FA value of the left anterior limb of the internal capsule **(B)**. The CAPS score was negatively correlated with the FA value of left anterior corona radiata **(C)** (*P* < 0.05, not corrected). PTSD, posttraumatic stress disorder; CAPS, Clinician-Administered PTSD Scale; FA, fractional anisotropy.

The results revealed a significant negative correlation between FA values in a cluster of the left posterior limb of the internal capsule, left anterior limb of the internal capsule, left anterior corona radiata, and the CAPS scores (*P* < 0.05, not corrected) (Figure 2). However, these significant correlation results did not survive after correction for multiple comparisons.

## Discussion

This study demonstrated that all typhoon-exposed individuals had white matter alterations, especially in the frontal fibers within the frontostriatal including corona radiata and internal capsule. Additionally, aberrant white matter alterations may be associated with the severity of PTSD symptoms.

Fractional anisotropy could be used as an indicator of white matter integrity. It is affected by many factors such as intra- and extracellular volume, fiber coherence, axonal density, myelination, membrane permeability, and partial volume effects, and thus, we should be cautious about the interpretation of FA alterations in white matter (Jones et al., 2013). MD measures the mean water diffusion rate. It is regarded as a metric of brain maturation and/or injury. Additional axial and radial diffusivities are helpful in better understanding the white matter microstructure changes. AD assesses the rate of water diffusion along the longitudinal axis and is regarded as an axonal marker (Alves et al., 2015). RD is associated with myelination (Bennett and Madden, 2014; Alves et al., 2015).

This study found that patients with PTSD demonstrated higher FA values in the superior longitudinal fasciculus. Superior longitudinal fasciculus is the main association fiber tract connecting cortical areas of the frontal, parietal, temporal, and occipital lobes. It is involved in visual aspects of goal-related motor activity, attention, and vigilance (Aschbacher et al., 2018). Altered superior longitudinal fasciculus FA values have been found in patients with PTSD in a meta-analysis (Daniels et al., 2013). Mitzy et al. (Kennis et al., 2015) also revealed a significant decrease over time in the superior longitudinal fasciculus FA of patients with remitted PTSD, indicating that the decreased FA values might be associated with the relief of symptoms. In summary, the superior longitudinal fasciculus alterations were associated with PTSD.

The increased FA in anterior corona radiata was in line with previous study (Aschbacher et al., 2018), which found increased FA in the anterior corona radiata in PTSD exposed to combat. The anterior corona radiata facilitates communication between the prefrontal and cingulate cortex with the thalamus and indirectly with the amygdala. The anterior corona radiata plays an important role in conflict resolution, attention, and emotion regulation (Niogi et al., 2010). In contrast, reduced FA was also found in the male combat veterans with comorbid PTSD and alcohol use disorders (AUD) compared with controls with AUD only. The participants and trauma type may account for the inconstancy. The higher FA values in this study might possibly be a result of increased myelination (reduced RD), which was associated with increased oligodendrocyte growth or migration (Li et al., 2016). Another possible explanation for the consistency of our results compared with some previous studies might be that we included both trauma-exposed group and nontrauma exposed as controls. Our findings indicate that the abnormalities of white matter in the anterior corona radiata may be associated with trauma exposure.

Increased FA values were found in the anterior and posterior limbs of the internal capsule in this study. The internal capsule mainly contains fibers projecting between the thalamus and cortex and includes optical fibers (Mori et al., 2008). Although its relationship to PTSD remains to be elucidated, it is reported to be susceptible to be influenced by PTSD (Morey et al., 2013). Importantly, the higher FA values in the internal capsule were correlated with CAPS scores, indicating that alterations in the internal capsule were associated with PTSD symptoms.

Unexpectedly, no significant differences were found in FA values and MD values between patients with PTSD and TEC. The small sample size, relatively short trauma exposure time may have accounted for this result. Decreased MD values were found in the posterior cingulum in the TEC group compared with the HC group, and no difference was found between PTSD and HC groups. The posterior cingulum provides the main route of communication between entorhinal and cingulate cortices, which is responsible for the effective integration of emotional and cognitive information (Fani et al., 2014). Disruptions in this area might have a detrimental effect on the affective and cognitive processes (Fani et al., 2012). Reduced microarchitectural integrity of the cingulum had been found in previous studies (Fani et al., 2012, 2014). Currently, the reason why the TEC group showed more significant decrease in MD compared with HC group remains unclear, which needs further investigation in the future. Additionally, most of the white matter alterations were found in both patients with PTSD and the TEC group. This indicated that the abnormalities might be possibly induced by trauma exposure.

Unexpectedly, the CAPS score was negatively correlated with FA value in multiple regions. However, no significant association was found between multiple diffusion indexes and multiple neuropsychiatric scores in different clusters of white matter tracts after correction for multiple comparisons. These might be related to the small sample size. A larger cohort should be included in the future to investigate the relationship between diffusion indexes and PTSD symptoms.

This study had several limitations that should be mentioned. First, the sample size was relatively small, and this was a cross-sectional study. A larger cohort with longitudinal follow-up should be considered in future studies. Second, this study only investigated typhoon-related PTSD, and the results might not be generalized to other etiologies of PTSD. Third, analysis tools and statistical methods will also affect the results. Finally, VBA also has its disadvantages to be solved, such as the arbitrariness of choosing spatial smoothness, the alignment of multiple FA images, and the standardization template of DWI images.

In conclusion, posttraumatic stress can lead to alterations of white matter in all typhoon-exposed individuals (with or without PTSD), especially in frontal fibers within the frontostriatal (corona radiata and internal capsule) and forceps of the corpus callosum. These findings offer more information for understanding the neural underpinnings of PTSD.

## Data Availability Statement

The datasets used during the current study are available from the corresponding author on reasonable request.

## Ethics Statement

The studies involving human participants were reviewed and approved by the Medical Research Ethics Committee of Hainan General Hospital and the Second Xiangya Hospital of Central South University. The patients/participants provided their written informed consent to participate in this study.

## Author Contributions

HC, RQ, and FC contributed to the conception and design of this study. HC, RQ, JK, JQ, and FC performed the statistical analysis and wrote the manuscript. HC, RQ, JK, QX, YZ, and GL performed the experiments. All authors contributed to manuscript revision and read and approved the submitted version.

## Funding

This study was supported by the National Nature Science Foundation of China (Grant numbers: 81971602, 81760308, 81801684, 81671672, 81871344, and 81701669), the Key Science and Technology Project of Hainan Province (Grant number: ZDYF2016156), and the Chinese Key Grant (Grant numbers: BWS11J063 and 10z026). This study was also supported by the Hainan Provincial Natural Science Foundation of China (Grant number: 818MS124) and the Program of Hainan Association for Science and Technology Plans to Youth R&D Innovation (Grant number: QCXM201919) the Nature Science Foundation of Jiangsu Province (Grant number BK20170368). This project was supported by Hainan Province Clinical Medical Center.

## Conflict of Interest

The authors declare that the research was conducted in the absence of any commercial or financial relationships that could be construed as a potential conflict of interest.

## Publisher's Note

All claims expressed in this article are solely those of the authors and do not necessarily represent those of their affiliated organizations, or those of the publisher, the editors and the reviewers. Any product that may be evaluated in this article, or claim that may be made by its manufacturer, is not guaranteed or endorsed by the publisher.
